# Tuning Reprocessing Temperature of Aliphatic Polyurethane Networks by Alkoxyamine Selection

**DOI:** 10.1021/acsapm.4c00840

**Published:** 2024-06-07

**Authors:** Fermin Elizalde, Vincent Pertici, Robert Aguirresarobe, Marta Ximenis, Giulia Vozzolo, Luis Lezama, Fernando Ruipérez, Didier Gigmes, Haritz Sardon

**Affiliations:** † POLYMAT, University of the Basque Country UPV/EHU, Joxe Mari Korta Center, Avda. Tolosa 72, 20018 Donostia-San Sebastian, Spain; ‡ Aix Marseille Univ, CNRS, ICR UMR 7273, 13397 Marseille, France; § Department of Inorganic Chemistry and BC Materials, University of the Basque Country UPV/EHU, E-48080 Bilbao, Spain; ∥ POLYMAT and Physical Chemistry Department, Faculty of Pharmacy, University of the Basque Country UPV/EHU, 01006 Vitoria-Gasteiz, Spain

**Keywords:** alkoxyamines, poly(urethane)s (PUs), covalent adaptable networks, reprocessing, thermosets

## Abstract

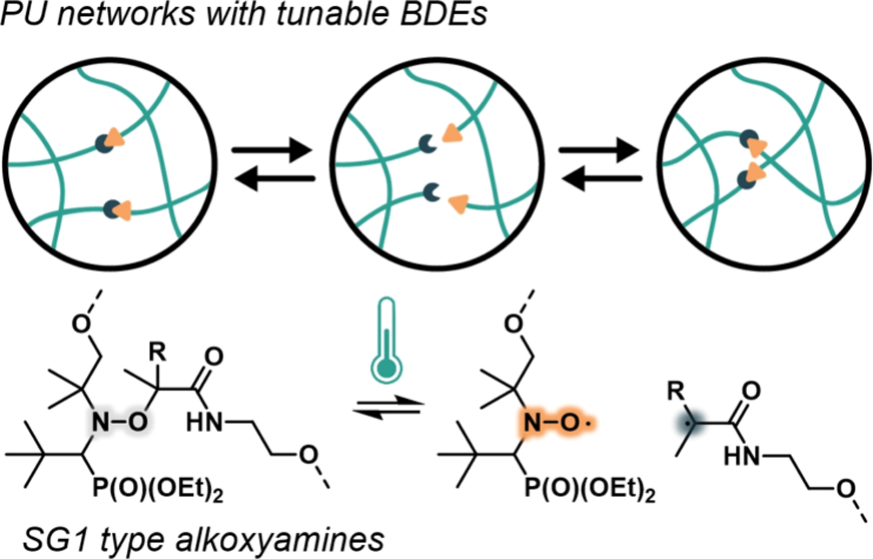

Recent studies have shown that the largest employed thermoset family, polyurethanes (PUs), has great potential to be reprocessed due to the dynamic behavior of carbamate linkage. However, it requires high temperatures, especially in the case of aliphatic PUs, which causes side reactions besides the desired exchange reaction. To facilitate the reprocessing of aliphatic PUs, in this work, we have explored the dynamic potential of alkoxyamine bonds in PU networks to facilitate the reprocessing under mild conditions considering their fast recombination ability. Taking advantage of the structural effect of the nitroxide and alkyl radicals on the dissociation energy, two different alkoxyamine-based diols have been designed and synthesized to generate PU networks. Our study shows that replacing 50 mol % of a nondynamic diol chain extender with these dynamic blocks boosts the relaxation times of the networks, enabling reprocessing at temperatures as low as 80 °C.

## Introduction

Classical thermosets have been employed for decades owing to their exceptional characteristics, including remarkable chemical resistance and mechanical properties. Nevertheless, the lack of recyclability of these permanently cross-linked networks has led to huge amounts of waste accumulation coming from discarded materials, creating a big impact on the environment.^1^ The introduction of specifically located dynamic linkages opened new opportunities for the reprocessing and recycling of these cross-linked materials, also described as covalent adaptable networks (CANs)^2−4^ without compromising the chemical resistance or thermal properties.

One of the most widely studied thermosets for recycling is the poly(urethane)s (PUs) family. Within the thermoset market, PUs are the most employed cross-linked materials. Some reports have shown that by properly selecting the catalyst and the isocyanate structure, these polymers can be reprocessable by transurethanization reaction^5^ following an associative or dissociative pathway.^6^ Indeed, this process is highly interesting from an industrial perspective as urethane groups are already present in the polymer, leading to the cheapest alternative to reprocessing this type of material. Unfortunately, this process has some drawbacks as relatively high temperatures are required to trigger the dynamic exchange, and important side reactions have been noticed especially in the case of aliphatic PUs.

For all of these reasons, orthogonal chemistries have been selected and introduced in conventional PUs to trigger exchange reactions in milder conditions.^5^ This strategy relies on introducing novel monomers with exchangeable bonds to conventional networks during polymer synthesis, enhancing their dynamic properties.^7,8^ According to the underlying mechanism, exchangeable bonds can be divided into dissociative, associative, and chain transfer reactions.^8^ Most important examples of such chemistries include Diels–Alder exchange reactions,^9^ transesterifications and vinylogous urethane exchange,^10^ or aromatic dichalcogenide rearrangements,^11−13^ among others. Depending on the chemistry included, the dynamic character can be activated upon different stimuli, such as light or redox, but the most common way is to use heat to trigger the exchange reaction.

**Scheme 1:**
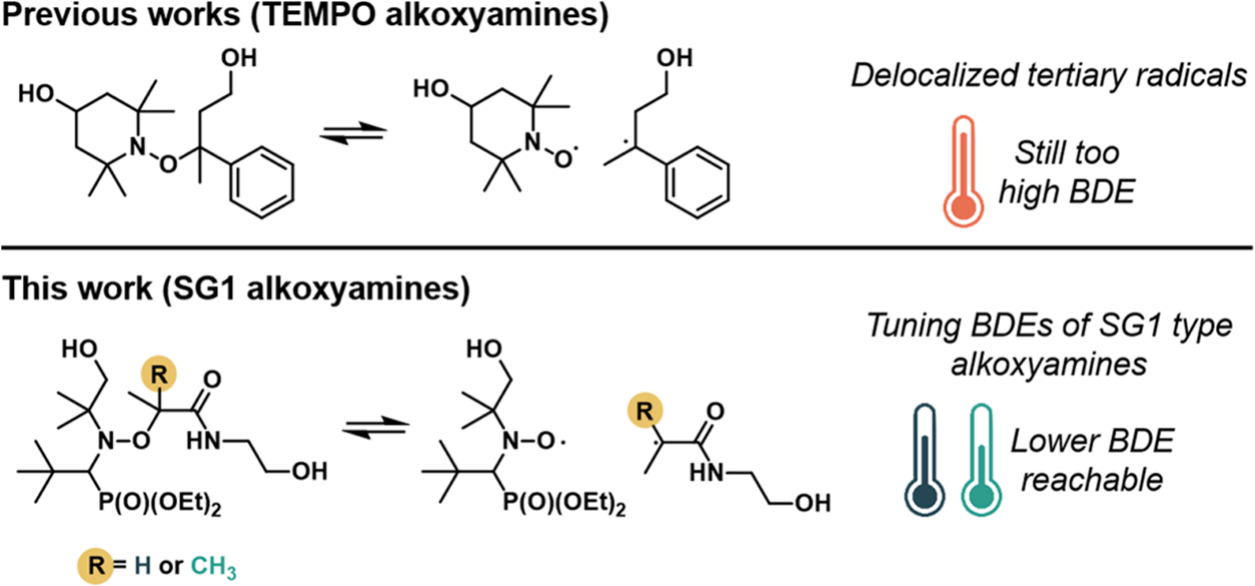
(a) Dissociation of 4-Hydroxy-1-(20-hydroxy-10-phenyl-10-methyl)ethyl-TEMPO (Diol), Reported in Previous Works. (b) Dissociation Equilibrium of the Two Alkoxyamine-Based Diols Studied in This Work

Among these groups, alkoxyamine chemistry has emerged as an interesting synthetic target, as these bonds can be reversibly cleaved into a persistent nitroxide radical and a transient carbon-centered radical, leading to fast exchanges. The fast recombination of thermally reversible C–ON bonds has been demonstrated for self-healing materials in previous works proving repeated cross-linking and de-cross-linking. Indeed, crack healing of PU is achieved by introducing 4-hydroxy-1-(20-hydroxy-10-phenyl-10-methyl)ethyl-TEMPO (diol) (Scheme 1a).^14^ Moreover, the dissociation energy of the alkoxyamines can be tuned through the molecular structure design. This concept has been particularly investigated in the field of controlled radical polymerization where it is essential to understand the reactivity of C–ON moieties.^15,16^ The regulation of the dissociation constant and homolysis temperature of polymeric materials carrying alkoxyamines has been extensively investigated in the literature especially to mediate controlled radical polymerization.^17^ Nevertheless, studies using alkoxyamines to tune the reprocessing temperature in PU networks are limited,^14,18^ and the molecular structures employed are 2,2,6,6-tetramethyl-1-piperidinyloxy (TEMPO)-based alkoxyamines,^19−22^ which exhibit higher dissociation temperatures compared to *N*-*tert*-butyl-*N*-[1-diethylphosphono-(2,2-dimethylpropyl)] nitroxide, also referred as SG1-type nitroxide alkoxyamines.^23,24^ For this reason, we decided to research their dynamic behavior in depth.

In this work, two alkoxyamine-based diols named PV1 and PV2 were designed and synthesized to introduce a dynamic bond with tunable dissociation temperatures (Scheme 1b). We targeted different dissociation temperature ranges by small variations in their molecular structures. After the evaluation of their dissociation behavior, these alkoxyamine-based diols have been introduced as chain extenders in a conventional PU formulation. After confirming the insertion into the PU backbone, their effect on the dynamic behavior of aliphatic PUs has been investigated. This type of PU is considered highly challenging as some recent reports have shown its inability to be reprocessed even in the presence of catalysts.^25^ Finally, the subsequent reprocessing cycles of these materials have been evaluated.

## Experimental Section

### Reagents

Dibutyltin dilaurate (DBTDL, 95%), hexamethylene diisocyanate (HDI, ≥98.0%), triethylamine (TEA), 2-amino-2-methylpropan-1-ol, 2-bromopropionyl bromide (97%), and anhydrous tetrahydrofuran (THF, ≥99.9%) were purchased from Aldrich and used as received. 1,6-Hexanediol was purchased from Aldrich and dried prior to use. Pivaldehyde was purchased from TCI and was used as received. Copper, copper bromide, *m*-CPBA, ethanolamine, 2-bromo-2-methylpropionyl bromide (98%), and *N*,*N*,*N*′,*N*″,*N*′′-pentamethyldiethylenetriamine (PMDETA) were purchased from Fisher and used as received. Poly(propylene glycol) (PPG) (*M*_n_ = 3740 g/mol) was purchased from Bayer Materials Science and dried in an oven overnight prior to use. Ethyl acetate (reagent grade), chloroform (reagent grade), and hexane (96%) were purchased from Scharlab.

### Synthesis and Characterization of PV1 and PV2 Alkoxyamines

**Figure 1:**
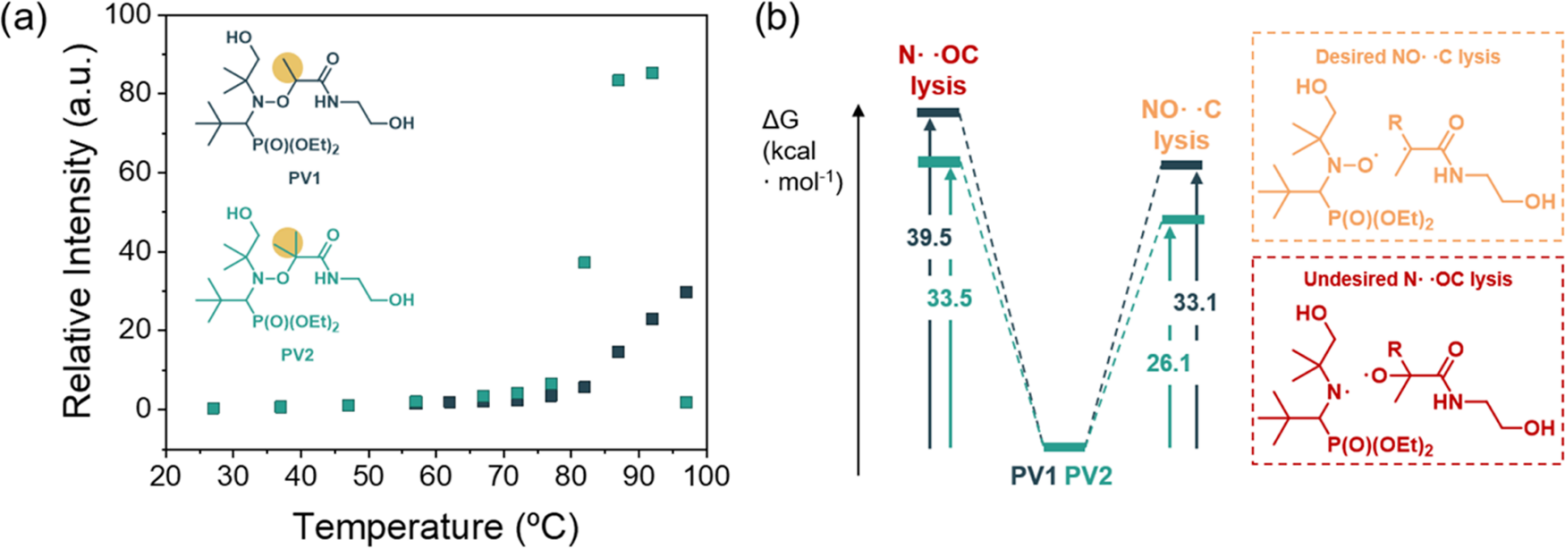
(a) Nitroxide radical intensities versus temperature obtained by electron paramagnetic resonance (EPR) for both PV1 and PV2 alkoxyamines. (b) Desired NO–C (yellow) bond cleavage and undesired N–OC (red) bond cleavage that can take place in alkoxyamines and the corresponding Gibbs free energies for PV1 (blue) and PV2 (green).

The alkoxyamines were prepared by following the steps reported in Scheme S1. The nitroxide part and the alkyl group were synthesized separately and coupled afterward. The nitroxide was synthesized based on the previously published pathways of Audran and Acerbis^26,27^ (Figure 1a).

#### Synthesis of 1-(Diethoxyphosphoryl)-2,2-dimethylpropyl 2-Hydroxy-1,1-dimethylethyl Nitroxide (**1**)

At 10 °C and under argon, 2-amino-2-methylpropan-1-ol (2.67 g, 30 mmol) was added dropwise to a solution of pivalaldehyde (2,2-dimethylpropanal; 2.33 g, 27 mmol). The mixture was heated up to reflux overnight, and the H_2_O was removed. Molecular sieves were added, and the solution was heated at 40 °C for 1 h. Diethylphosphonate (5.8 g, 42 mmol) was added at room-temperature (rt), and the mixture was heated at 40 °C for 6 days. The mixture was poured in CH_2_Cl_2_ and the precipitate was filtered off. The solution was acidified with 5% HCl solution to reach pH = 3 and then washed with CH_2_Cl_2_ (5 × 20 mL). The aqueous layer was basified with KHCO_3_ (pH = 8) and then extracted with CH_2_Cl_2_ (2 × 20 mL), the organic layer dried over NaSO_4_, and the solvent evaporated to get the product (amine) (3.58 g, 45%) as a colorless oil. *meta*-Chloroperbenzoic acid (5.38 g, 24 mmol) was added to a solution of the aminophosphonate (3.5 g, 12 mmol) in CHCl_3_ at 0 °C. After stirring the mixture for 24 h, it was diluted with CHCl_3_, and washed with aq. sat. NaHCO_3_ solution, dried, and concentrated *in vacuo*. Column chromatography of the residue gave **1** as an orange powder.

The alkyl groups (2-bromo-*N*-(2-hydroxyethyl)-propanamide in case of PV1 and 2-bromo-*N*-(2-hydroxyethyl)-2-methylpropanamide for PV2 (**2a** and **2b**, respectively)) were synthesized based on a procedure described by Huang and Chang.^28^ Viscous oils. **2a**, 3.6 g, (55%) and **2b**, 3.9 g, (58%).

#### Radical Coupling to Render PV1 and PV2

The radical coupling was performed between the nitroxide and the alkyl group to obtain the final product,^29^ following an already reported procedure.^16,30^ To a Schlenk flask was added **2a** or **2b**, 980.2 mg and 1.05 g (5 mmol); **1**, 1.86 g (6 mmol); copper powder, 381 mg (6 mmol); CuBr, 7.2 mg (0.05 mmol); PMDETA, 347 mg (2 mmol) in THF. The reaction solution was degassed, put under argon, and stirred for 6 h at rt. After solvent evaporation, the crude product was loaded onto a silica column (from 100% EtOAc to EtOAc/MeOH 85:15) and obtained as colorless fractions. Yield of PV1 was 84% (1.8 g) and yield of PV2 was 86% (1.89 g) as white solids.

#### Diethyl (1-((1-Hydroxy-2-methylpropan-2-yl)((1-((2-hydroxyethyl)amino)-1-oxopropan-2-yl)oxy)amino)-2,2-dimethylpropyl)phosphonate (**PV1**)

^1^H NMR (300 MHz, DMSO) δ 8.19 (t, *J* = 5.6 Hz, 1H), 4.90 (t, *J* = 5.5 Hz, 1H), 4.69–4.59 (m, 2H), 4.47–4.27 (m, 1H), 4.21–3.99 (m, 2H), 3.50–3.39 (m, 6H), 3.28–2.95 (m, 2H), 1.35 (d, *J* = 6.8 Hz, 3H), 1.28–1.13 (m, 8H), 1.12–0.88 (m, 23H). ^13^C NMR (75 MHz, DMSO) δ: 173.44, 80.29, 69.05, 67.55, 65.46, 61.49, 59.74, 41.36, 29.44, 23.51, 22.35, 18.88, 16.35.

#### Diethyl (1-((1-Hydroxy-2-methylpropan-2-yl)((1-((2-hydroxyethyl)amino)-2-methyl-1-oxopropan-2-yl)oxy)amino)-2,2-dimethylpropyl)phosphonate (**PV2**)

^1^H NMR (300 MHz, DMSO) δ 8.01 (t, *J* = 5.6 Hz, 1H), 5.32 (t, *J* = 5.8 Hz, 1H), 4.66 (t, *J* = 5.5 Hz, 1H), 4.23–3.91 (m, 4H), 3.56–3.39 (m, 3H), 3.31–2.96 (m, 4H), 1.49 (s, 6H), 1.26 (dt, *J* = 13.8, 7.0 Hz, 7H), 1.17–0.93 (m, 17H). ^13^C NMR (75 MHz, DMSO) δ: 175.34, 84.95, 69.94, 68.18, 67.41, 65.72, 61.47, 60.03, 59.59, 41.48, 29.41, 29.34, 26.16, 24.73, 24.69, 23.53, 16.44, 16.37, 16.11, 16.03.

### Synthesis of Reprocessable PU Thermosets

Synthesis of aliphatic tris-isocyanate-terminated prepolymer. The reprocessable PU thermosets were prepared as recently described. Briefly, PPG and HDI were mixed in a nitrogen atmosphere to obtain a tris-isocyanate-terminated prepolymer Yield 92 g, 90%.^25^ Synthesis of aliphatic cross-linked PU films (Blank, PU–PV1, and PU–PV2). 1,6-Hexanediol (127 mg, 1.06 mmol) previously dissolved in 0.2 mL of anhydrous THF was mixed with aliphatic tris-isocyanate-terminated prepolymer (3 g, 0.71 mmol) and stirred vigorously. 2 mol % of NCO content of DBTDL was directly added to the mixture. The mixture was degassed under vacuum and was placed in an open mold. The curing was carried out at 80 °C for 2 h. As an example of the synthesis of PU-containing 50% of PV1 or PV2 alkoxyamines in the polymer networks, PV1 (225.8 mg, 0.53 mmol) or PV2 (233.5 mg, 0.53 mmol) was dissolved in 1 mL of THF and added with 1,6-hexanediol (63.5 mg, 0.53 mmol) to the aliphatic tris-isocyanate terminated prepolymer (3 g, 0.71 mmol) and stirred vigorously. 2 mol % of NCO content of DBTDL was directly added to the mixture.

### Characterization Methods

Fourier transform infrared (FTIR) spectroscopy and nuclear magnetic resonance (NMR) were used to characterize the polymers similar to previous reports.^25^

A Nicolet 6700 FTIR, Thermo Scientific, Inc., and Bruker Avance DPX 300 and Bruker Avance 400 spectrometers were used.

Thermogravimetric analyses (TGA), stress relaxation experiments, and dynamic mechanical thermal analysis (DMTA) were performed to analyze the mechanical and thermal properties of the material. To do so, a TGA/Q500 TA Instruments ARES rheometer (Rheometrics) and Triton 2000 DMA (Triton Technology) were used as recently reported.^31^

### Electron Paramagnetic Resonance

Measurements were performed at room temperature using a Bruker ELEXSYS E500 spectrometer operating at the X-band.^32^ Typical instrument settings were as follows: center field, 3305 G; scan range, 60 G; receiver gain, 30 dB; time constant, 5.12 ms; modulation amplitude, 1.0 G; microwave power, 2 mW; accumulated scans, 5.

### Density Functional Theory (DFT) Calculations

All geometry optimizations were carried out within density functional theory (DFT) using the same functional that we used in a previous publication.^25^ See more details in ESI.

## Results and Discussion

As mentioned in the Introduction section, one of the main drawbacks of TEMPO-based alkoxyamines in PU reprocessing is the high temperatures required for reprocessing, which often lead to material degradation. To address this issue, our approach in this work focuses on utilizing SG1-type nitroxides to mitigate these harsh conditions.^33^ To understand how small modifications in the molecular structure of such nitroxides affect the radical exchange rate of these species, PV1 (R = H) and PV2 (R = CH_3_) alkoxyamine-based diols have been prepared. The core of the alkoxyamine is based on SG1-type nitroxide and our rational design is based on changing the nature of the released alkyl part as it has been previously reported that could impact their dissociation temperature.^23^ The alkoxyamines were prepared in a two-step process, where the nitroxide part and the alkyl group were synthesized separately and coupled afterward. The nitroxide was synthesized based on the previously published pathway of Acerbis et al. (Scheme S1).^27^ The alkyl group (2-bromo-*N*-(2-hydroxyethyl)-propanamide) in case of PV1 and 2-bromo-*N*-(2-hydroxyethyl)-2-methylpropanamide for PV2 were synthesized based on a procedure described by Huang and Chang.^28^ Then, radical coupling was performed between the nitroxide and the alkyl group to obtain the final product,^29^ following an already reported procedure.^16,30^

Both alkoxyamines have been fully characterized by nuclear magnetic resonance (^1^H NMR, ^13^C NMR) and correlated spectroscopy (COSY) experiments (Figures S1–S3). The main difference between ^1^H NMR spectra is evidenced by signal number 8 relative to the R group. In the PV1 alkoxyamine spectra, the signal integration is three (3) which is characteristic of the methyl group, while in PV2 alkoxyamine, it is close to 6, as expected for the 2 methyl substituents. The PV1 alkoxyamine spectrum also displays the proton bound to the carbon atom adjacent to the oxygen which corresponds to the signal number 15. The absence of this peak in PV2 spectra also confirms the molecular structure of this alkoxyamine. These characterizations were corroborated by COSY experiments (Figure S2).

After characterization of the alkoxyamine-based diols, we evaluated the dissociation ability of the alkoxyamine using electron paramagnetic resonance (EPR) measurements. To establish the homolytic dissociation, we determined the radical nitroxide concentration upon heating of both PV1 and PV2 alkoxyamines. Both EPR measurements showed an increasing signal associated with the appearance of nitroxide species (Figures 1a and S4). The EPR evaluation allowed us to ascertain that these radicals are associated with the nitroxide groups due to NO–C bond cleavage. As expected, there is a strong dependence of the formed radicals on the chemical structure. PV2 alkoxyamine undergoes a radical dissociation under relatively mild conditions, as it shows a noticeable nitroxide signal increase near 80 °C. In contrast, PV1 alkoxyamine requires higher temperatures to reach the same degree of dissociation. This effect is related to both the higher stabilization of the generated alkyl radical and the higher steric hindrance within the starting alkoxyamine.^34^ Nevertheless, PV2 shows a remarkable drop in the nitroxide EPR signal above 90 °C, which will be discussed in more detail in a later section.

To get a better understanding of the dissociation mechanism, density functional theory (DFT) calculations have been performed. It is important to notice that two different dissociations are plausible in the case of alkoxyamines; the desired dissociation of NO–C bond (providing relatively stable nitroxide radical ideal for reprocessing) or the dissociation of N–OC bond (providing highly reactive aminyl and alkoxyl radicals not suitable for reprocessing). It should be pointed out that DFT calculations show that in both PV1 and PV2, the N–OC bond is stronger than the NO–C bond (39.5 and 33.5 kcal/mol compared to 33.1 and 26.1 kcal/mol for PV1 and PV2, respectively), providing the desired product. When comparing the dissociation energies of PV1 and PV2, the DFT calculations are in agreement with the experimental data as they show that the lowest bond dissociation energy has been obtained for PV2 structure (26.1 kcal/mol) compared to PV1 (33.1 kcal/mol) suggesting that this structure will be more dynamic at milder conditions (Figure 1b).

**Figure 2:**
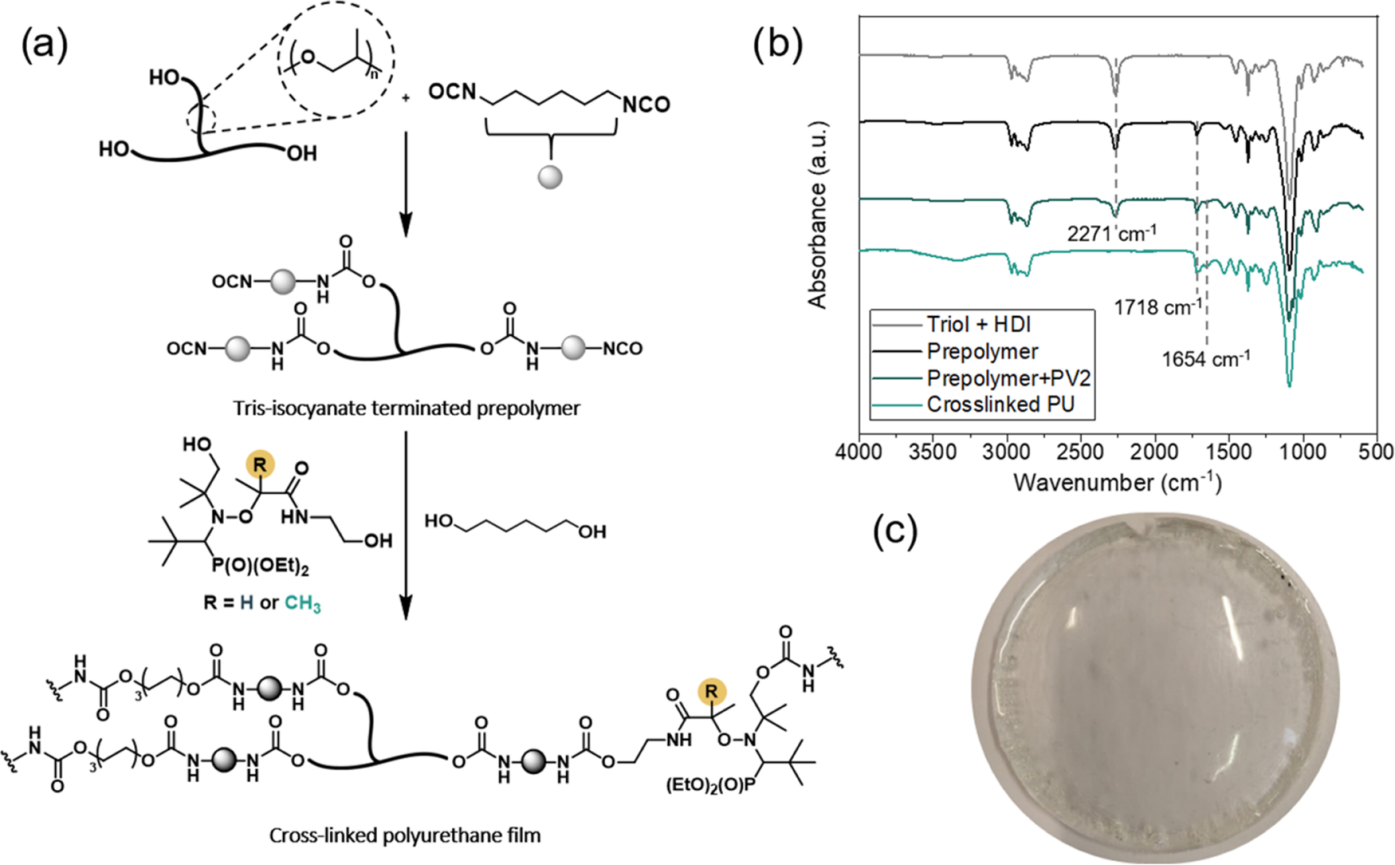
(a) Synthetic procedure for obtaining aliphatic cross-linked PU with different chain extender contents of either PV1 or PV2 alkoxyamine and 1,6-Hexanediol (HDO). (b) Representative FTIR spectra of the synthesized prepolymer with HDI, after the addition of 50 mol % of PV2 alkoxyamine and 50 mol % of 1,6-hexanediol and final PU film cross-linked for 1 h at 70 °C. (c) Representative film of the obtained material.

Once we synthesized and studied the radical dissociation of PV1 and PV2, we introduced them in a reference PU thermoset structure to render dynamic materials, as shown in Figure 2a (see the Experimental Section). First, we synthesized a tris-isocyanate-terminated prepolymer by reacting commercially available poly(propylene glycol) (PPG) with an average molecular weight of 3.7 kDa, with hexamethylene diisocyanate (HDI). The isocyanate selection was based on taking into account our previous research which demonstrated that aliphatic PU thermosets do not undergo transcarbamoylation exchange reactions that could allow these materials to stress relax fast at temperatures as low as 120 °C.^25^ The reference material was obtained in a second step by adding 1,6-hexanediol (HDO) to the prepolymer. After complete curing (60 °C overnight), a cross-linked aliphatic PU film was obtained. Dynamic thermoset films were synthesized by replacing different amounts of the aforementioned chain extender (HDO) equivalents with PV1 and PV2 alkoxyamines with different molar ratios. For all cases, 2 mol % of dibutyltin dilaurate (DBTDL) was added before the curing step. This catalyst was employed for its ability to accelerate the synthesis of PU networks. Additionally, it has been demonstrated that there is no dynamic behavior in aliphatic PU thermosets.

To follow both synthetic steps (prepolymer formation and the subsequent curing process), FTIR spectroscopy was employed. As can be seen in the synthesis of dynamic aliphatic PU networks with 50% of PV2 alkoxyamine (Figure 2b) and reference HDO PU (Figure S5), the isocyanate stretching band at 2271 cm^–1^ completely disappeared and new bands corresponding to the formation of the urethane linkage appeared at 1718 cm^–1^. The band at 1654 cm^–1^ appeared due to the stretching of the N–CO group of alkoxyamines. In all of the cases, the proposed synthetic pathway led to homogeneous transparent polymers for alkoxyamine-based PU (Figure 2c).

### Effect of the Chemical Structure of Alkoxyamines and Concentration on the Dynamic Behavior of Aliphatic PU Thermosets

**Figure 3:**
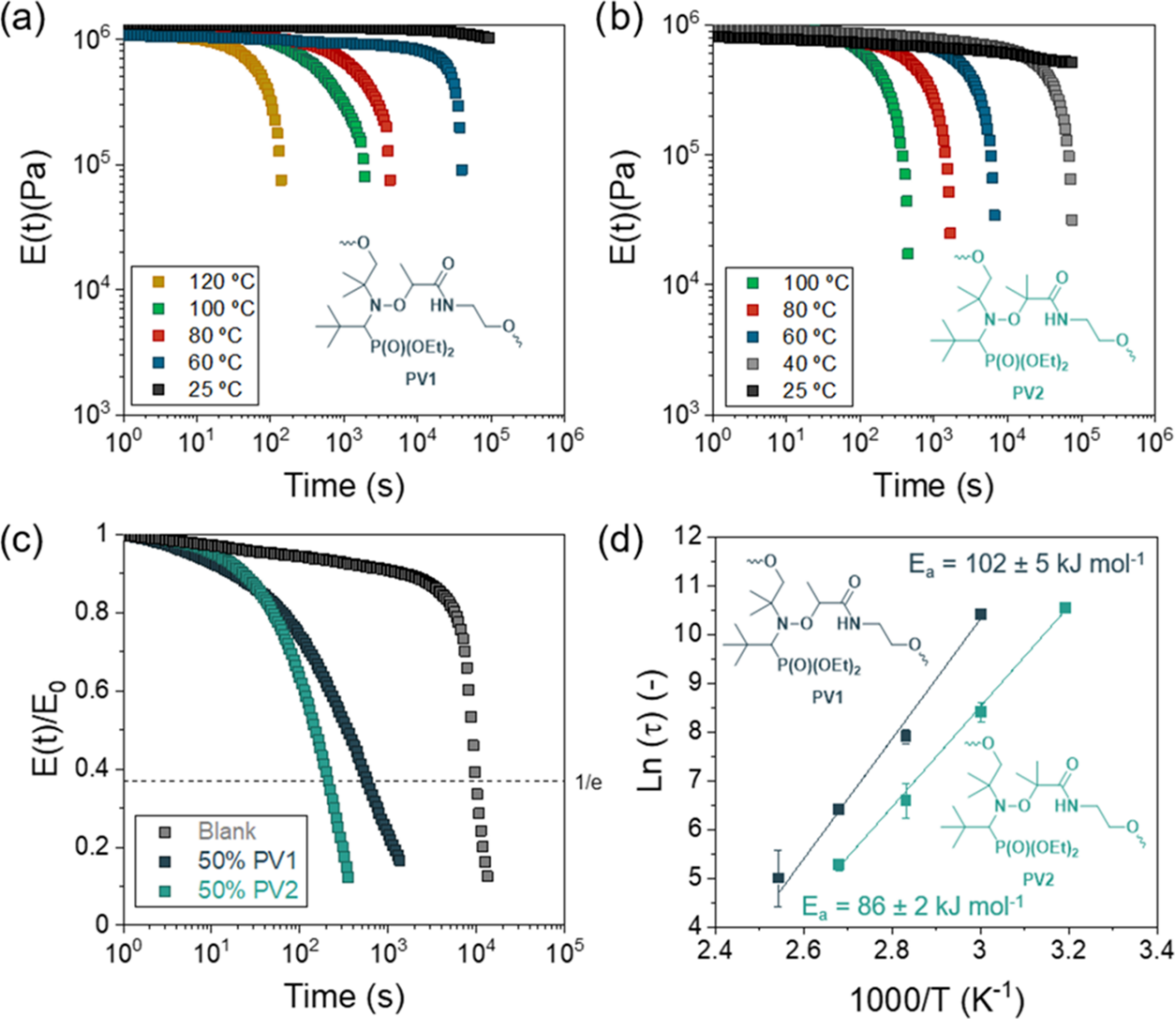
(a) Stress relaxation measurements for a representative network 50/50 (PV2/HDO) performed at 100, 80, 60, 40 and 25 °C. (b) Stress relaxation measurements for a representative network 50/50 (PV1/HDO) performed at 120, 100, 80, 60 and 25 °C. (c) Influence of the alkoxyamine nature in the relaxation time. Comparison of relaxation times at 100 °C for pristine PU and both 50% PV1- and PV2-containing PU networks. (d) Arrhenius plot of characteristic relaxation time of each cross-linked PU and their corresponding activation energies (*E*_a_).

After the films were characterized, the dynamic behavior of the different films was analyzed by stress relaxation measurements in tension mode at different temperatures and compared with the blank PU without any dynamic bond. In both cases, 50 mol % on 1,6-hexanediol has been exchanged by the corresponding alkoxyamine. As expected, the increase in temperature fastened the relaxation of the material (Figure 3a,b, for PV1 and PV2, respectively). Materials containing alkoxyamines showed a fast relaxation of the relaxation modulus *E*(*t*) with time even at temperatures below 100 °C. However, it is remarkable that at room temperature we did not observe any relaxation of the cross-linked PU film (black traces). At this condition, we surmise that the dissociation equilibrium of the alkoxyamine/nitroxide is shifted totally to the alkoxyamine state. In addition, the relaxation behavior is highly dependent on the alkoxyamine nature. Fast relaxation times have been obtained at 100 °C, temperatures above the estimated dissociation temperature of both PV1 and PV2 alkoxyamines. As expected from EPR measurements, the higher dissociative character of PV2 leads to a faster relaxation in the synthesized PU network (see comparison in Figure 3c). It is to be noted that the unfunctionalized sample shows a decay in the relaxation modulus at very long times (∼10^4^ s), caused by partial degradation of the network, as we recently reported.^25^ This effect is also reflected in the Arrhenius activation energy (Figure 3d), which shows a higher value for PV1 (102 kJ/mol) than for PV2 (86 kJ/mol).

Additionally, the effect of the alkoxyamine concentration on the dynamic character of PU thermosets was analyzed by using PV2 as a reference. As shown in Figure S5, the fast homolytic cleavage of alkoxyamine can produce the relaxation of the polymer network by just replacing 10 mol % of the total chain extender in the material. Even at this low concentration, relaxation occurs below 980 s at 100 °C. In addition, the activation energy shows a slight dependence on the alkoxyamine concentration (from 100 to 80 kJ/mol). The difference in the activation energy can be related to the different mechanical performances of the polymer network (*E*(*t*)0 values) when changing the alkoxyamine concentration.

### Reprocessing Capabilities of Alkoxyamine-Based PU

**Figure 4:**
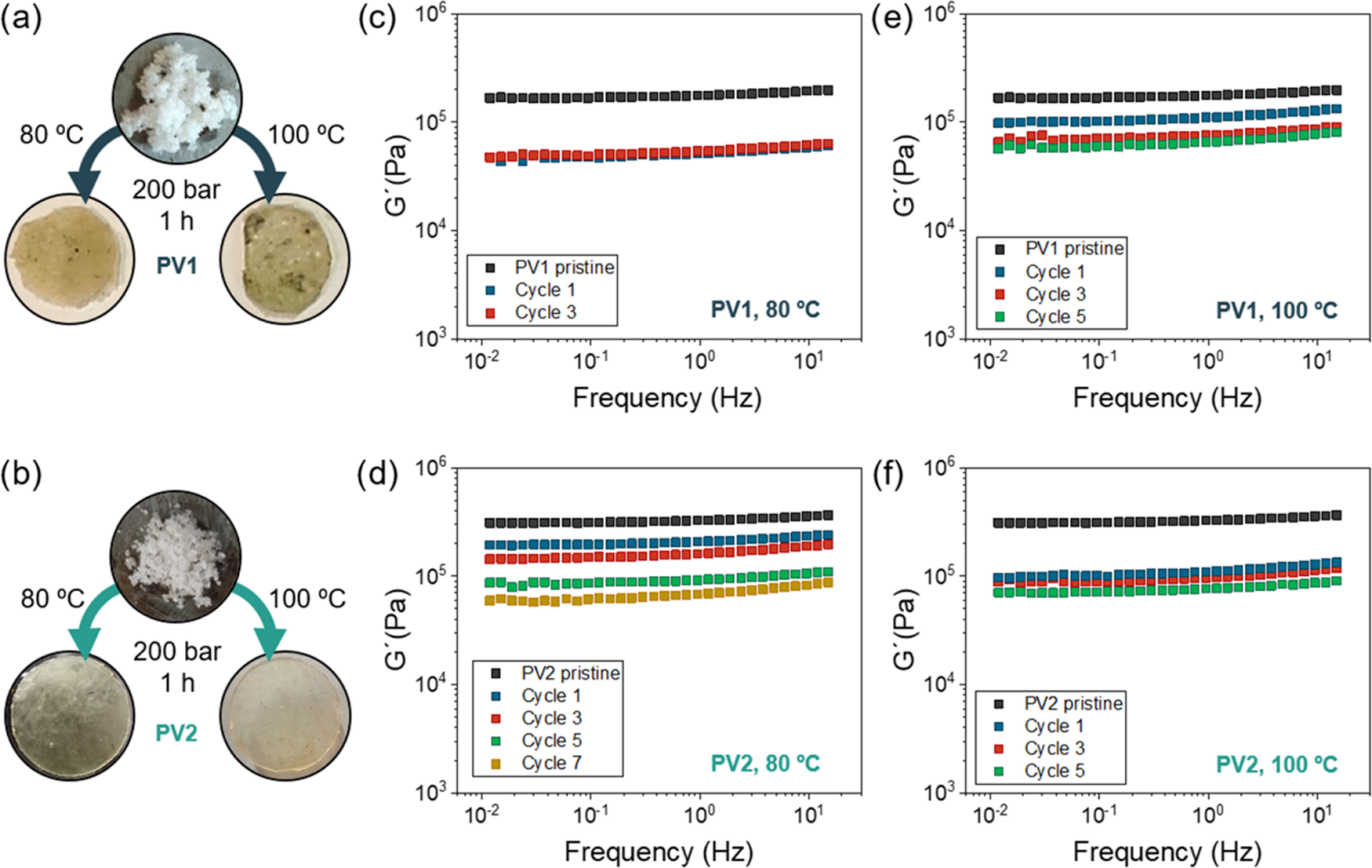
Reprocessing characteristics for the 50/50 (PV/HDO) samples: (a) PV1 and (b) PV2. *G*′ vs frequency scans after several reprocessing cycles at (c, d) 80 °C and (e, f) 100 °C for PV1- and PV2-based systems.

Based on the stress relaxation results, the reprocessing capabilities of materials were tested (Figure 4). Thus, the above-mentioned dynamic networks were ground and placed in a thin metal layer mold and introduced in a hot press for reprocessing at 80 or 100 °C for 60 min with a pressure of 200 bar. As can be seen in Figure 4a,b, the material was introduced as a white powder, and after the reprocessing, disks with a diameter of around 8 mm were obtained. To evidence the radical dissociative mechanism of alkoxyamines, a reprocessing attempt was performed for a representative PU network containing 50% PV2 in the presence of tributyl tin hydride, a radical inhibitor (Figure S7). The material could not be reprocessed, thus evidencing the radical dissociative mechanism for the exchange.

The mechanical characteristics of the polymer networks were analyzed by small-amplitude oscillatory shear experiments in frequency sweeps (Figure 4c–f). In the case of the PV2-containing PU network, the best-reprocessed material was obtained at 80 °C (Figure 4d) as the obtained plateau modulus is close to that of virgin material. However, as the number of reprocessing cycles increases, the obtained plateau modulus for the material decays. This behavior is more noticeable for 100 °C (Figure 4f) and the modulus drop is observed even in the first reprocessing. A similar behavior is observed for PV1 at 100 °C (Figure 4e). This behavior is attributed to degradation processes that will be discussed later on. It is noticeable that in the case of the PV1-containing PU network, at 80 °C (Figure 4c), the films obtained are not homogeneous and not fully sealed as can be seen in the picture (Figure 4a). We surmise that this effect can be attributed to a temperature effect, which is still too low to effectively open the majority of the alkoxyamine linkages. This would be in line with the radical intensities observed in EPR experiments at 80 °C. Nonetheless, the absence of a significant decrease in storage modulus following a single cycle of reprocessing is undeniably a favorable result.

The mechanical properties of PU-containing 10% PV2 alkoxyamine were also investigated by dynamic mechanical analysis (DMA). The results of this analysis are presented in Figure S13. The DMA test reveals that changes in the network occur during reprocessing. Also, the EPR experiments (Figure S10) reveal an increase in the radical species formation within the network. Overall, these experiments support a dissociative exchange mechanism. The absence of a significant decrease in the storage modulus following a single cycle of reprocessing is undeniably a favorable result.

### Limitations of Synthesized Alkoxyamine-Based PU Thermosets

**Figure 5:**
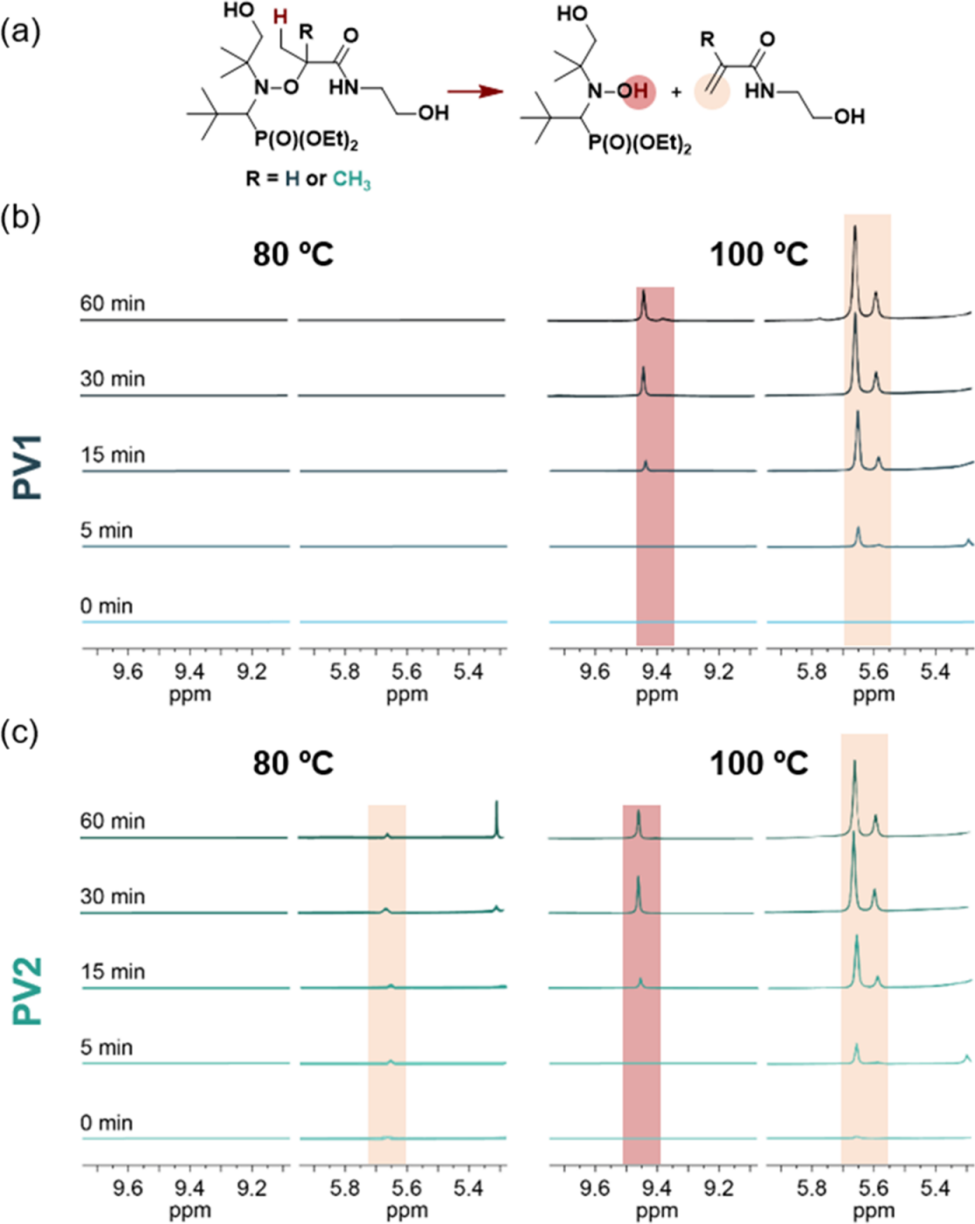
(a) Proposed side elimination reaction. ^1^H NMR spectra at different times for (b) PV1 and (c) PV2 alkoxyamines preheated at 80 °C (left) and 100 °C (right), respectively.

As observed in the reprocessing experiments, the mechanical properties of films decay as the number of reprocessing cycle number increases. In addition, according to EPR measurements, PV2 alkoxyamine suffers a sudden drop in nitroxide concentration above 90 °C (Figure 1a). We surmise that undesired side reactions may take place as has been previously described for SG1-type nitroxide alkoxyamines.^24,35^ Indeed, intramolecular H atom transfer (HAT) can lead to the formation of the corresponding hydroxylamine and alkene products. Thus, to get more insights into this behavior, we performed ^1^H NMR experiments for both alkoxyamines at 80 and 100 °C (Figure 5). Thus, several aliquots at different times (5, 15, 30, and 60 min) were taken to assess the degradation behavior of each alkoxyamine-diol.

Experiments performed on PV1 show no side reactions at 80 °C; however, the immediate appearance of characteristic signals of HAT is evidenced at 100 °C, corresponding to the hydroxylamine proton (around 9.4 ppm) and the alkene protons (around 5.6 ppm). For PV2, analogue signals can be observed, in this case at both temperatures. These results are in line with the EPR measurements, which evidence the radical formation for each alkoxyamine, being possible at lower temperatures for PV2. In both cases, the long exposure to temperature not only allows for radical dissociation but also seems to trigger the side HAT reaction, which irreversibly leads to the corresponding hydroxylamine and alkene. The translation of this phenomenon to the alkoxyamine-based PU networks would explain the decrease in the mechanical properties observed by a frequency sweep upon reprocessing cycles.

## Conclusions

In summary, it has been demonstrated that the introduction of different alkoxyamines can tune the stress relaxation of aliphatic PU networks and, therefore, become a key factor for the reprocessing of these materials at low temperatures. Although both alkoxyamine-based diols have shown high dissociative behavior at 100 °C, their stability is compromised as shown by EPR and ^1^H NMR measurements. Nevertheless, PV2 alkoxyamine is the most interesting and stable structure for the final reprocessing of these materials at 80 °C. Overall, this work has proved the effective introduction of SG1 alkoxyamine-based diols for the reprocessing of aliphatic PU networks at temperatures as low as 80 °C, avoiding undesired secondary reactions that could occur from dissociative transcarbamoylation reactions at higher temperatures. However, as shown by the experimental data, competing reactions still exist. Thus, the future design of alkoxyamine-based diols should consider both the fast reprocessing and the reduction of side reactions to allow for the reprocessing of the material several times.

## Supporting Information Available

Synthetic scheme, ^1^H NMR and COSY spectra, EPR of PV1 and PV2, FTIR characterization of HDO-based PU, stress relaxation measurements of PU films containing different % of PV2 and corresponding Arrhenius plot, and reprocessing attempt in the presence of a radical inhibitor for PV2-PU film (PDF)

## Abbreviations

PU: polyurethane
CANs: covalent adaptable networks
HAT: H atom transfer
