# Supplementary material for: Tuning Reprocessing Temperature of Aliphatic Polyurethane Networks by Alkoxyamine Selection
Source: ACS Appl Polym Mater. 2024 Jun 7;6(12):7057–65. doi: 10.1021/acsapm.4c00840 (PMC11217918; doi:10.1021/acsapm.4c00840)
Supplement: Supplementary file 1 — ap4c00840_si_001.pdf [file ap4c00840_si_001.pdf]

Supporting Information

Tuning reprocessing temperature of aliphatic polyurethane networks by alkoxyamine selection

Fermin Elizalde,[1] Vincent Pertici,[2] Robert Aguirresarobe,[1] Marta Ximenis,[1] Giulia Vozzolo,[1] Luis Lezama,[3] Fernando Ruipérez,[4] Didier Gigmes[2] and Haritz Sardon[1*]

[1]POLYMAT, University of the Basque Country UPV/EHU, Joxe Mari Korta Center, Avda. Tolosa 72, 20018 Donostia- San Sebastian, Spain
[2]Aix Marseille Univ, CNRS, ICR UMR 7273, 13397 Marseille, France.
[3]Department of Inorganic Chemistry and BC Materials, University of the Basque Country UPV/EHU, E-48080 Bilbao, Spain
[4]POLYMAT and Physical Chemistry Department, Faculty of Pharmacy, University of the Basque Country UPV/EHU, 01006 Vitoria-Gasteiz, Spain

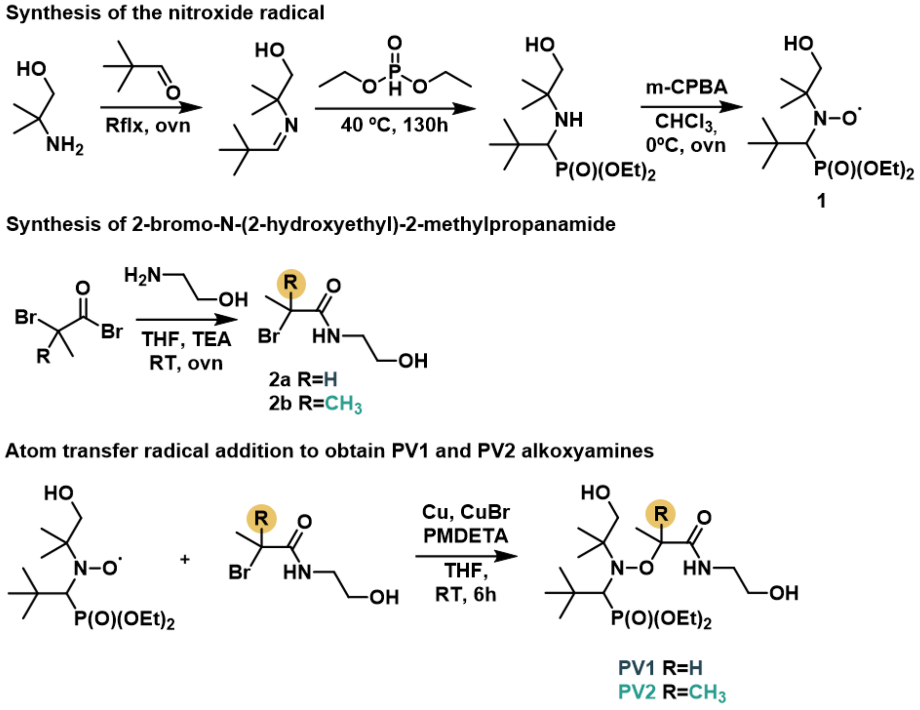

Scheme S1. Synthetic pathway towards PV1 and PV2.

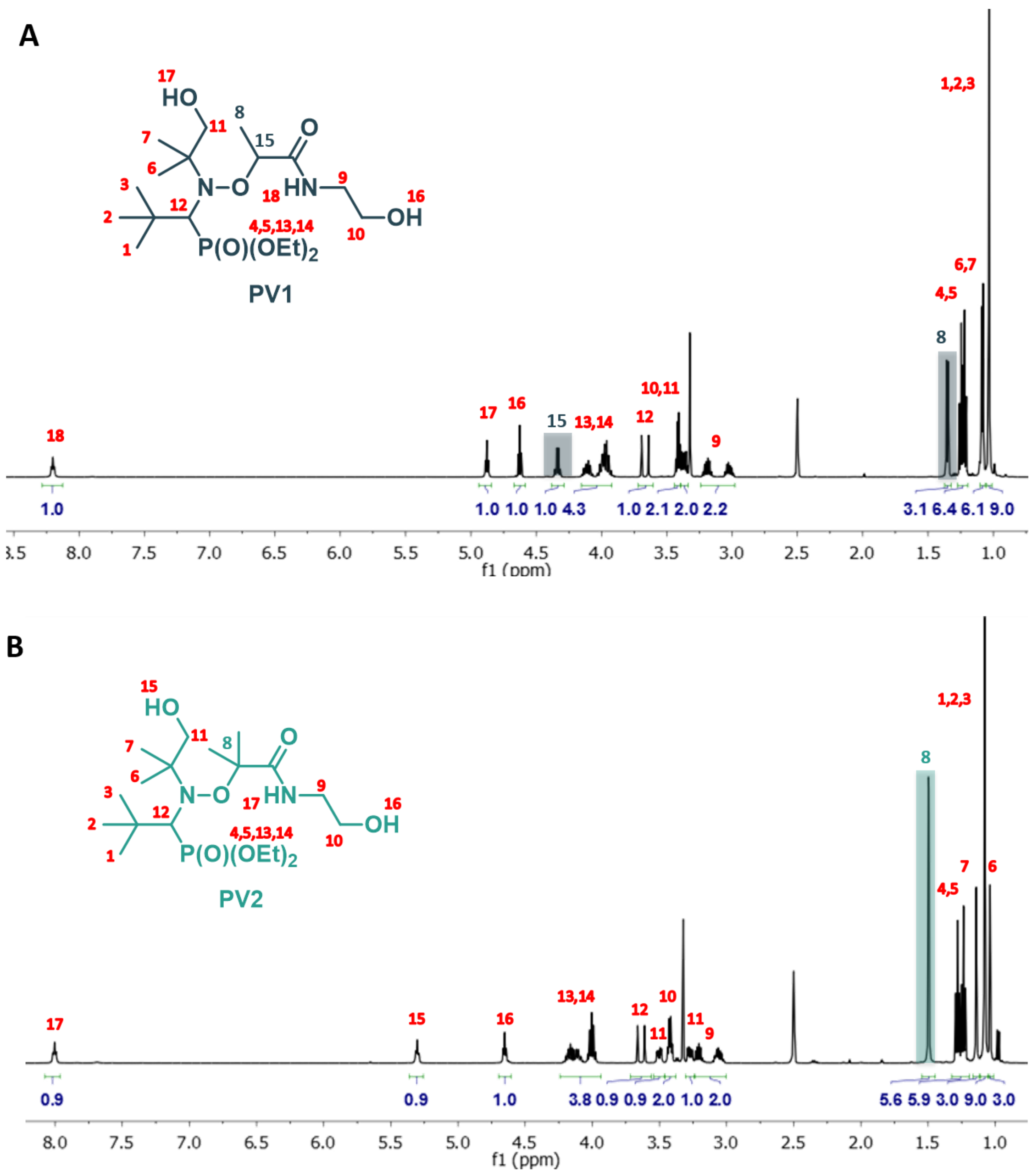

Figure S1. ^{1}H-NMR spectra of PV1 alkoxyamine (A) and PV2 alkoxyamine (B) recorded in DMSO-d_6.

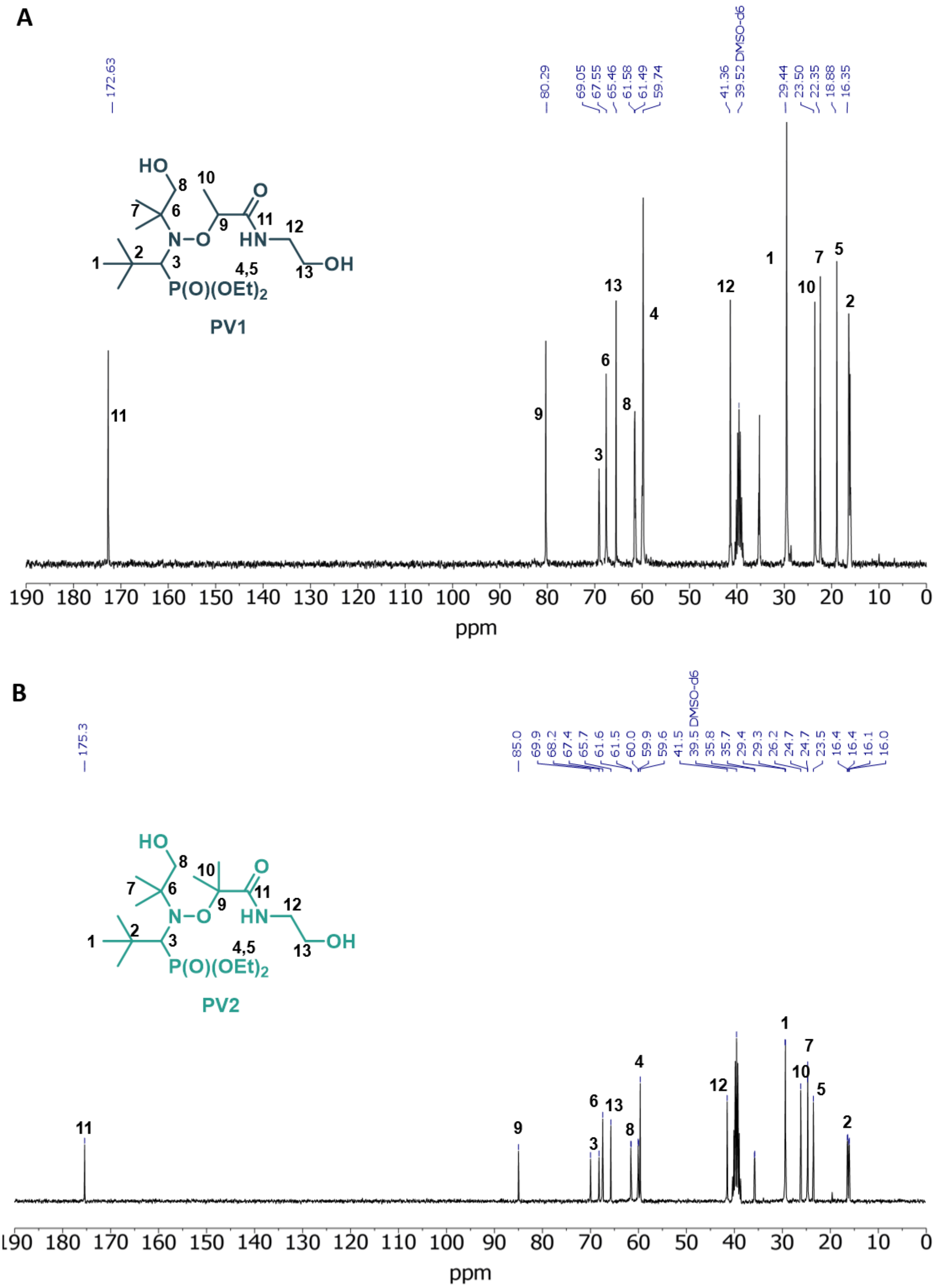

Figure S2. ^{13}C-NMR spectra of PV1 alkoxyamine (A) and PV2 alkoxyamine (B) recorded in DMSO-d_6.

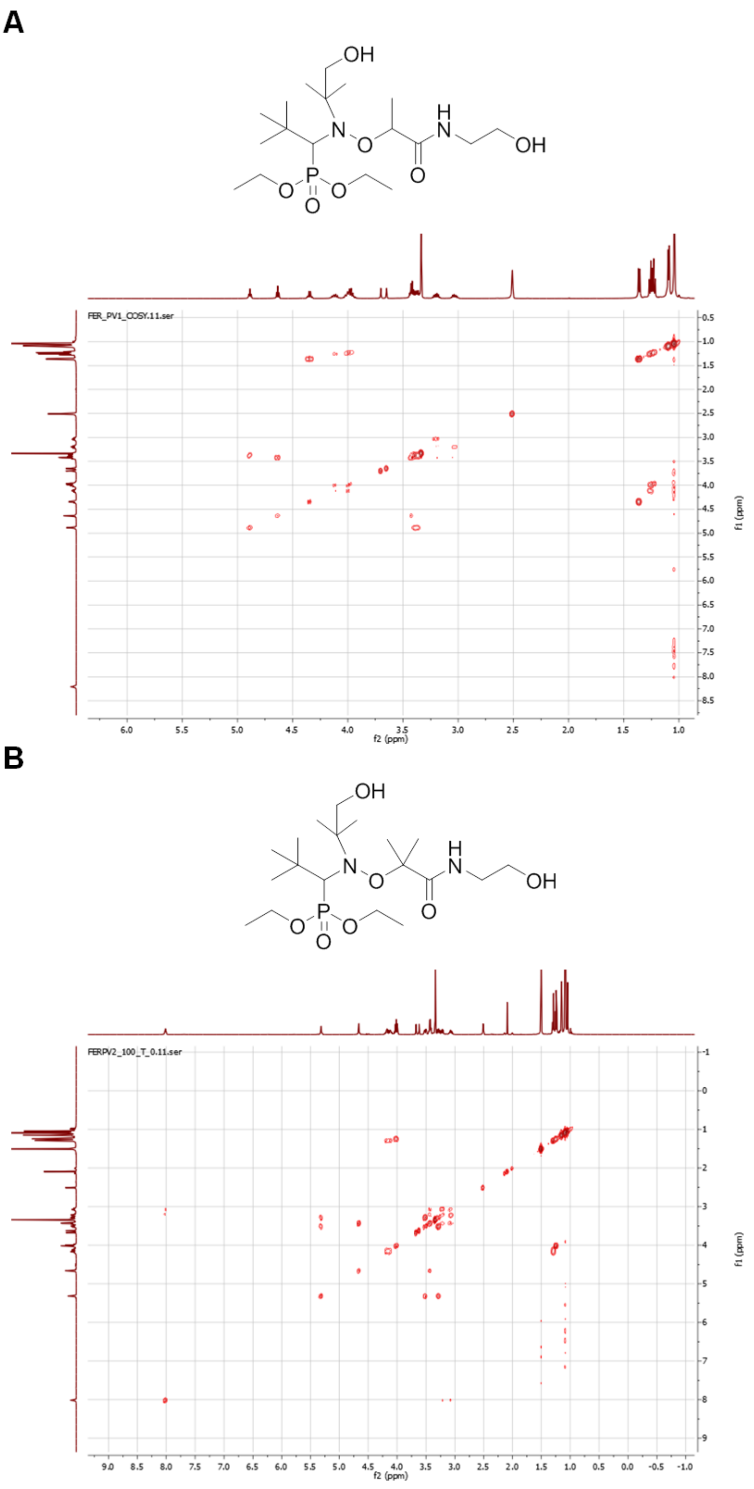

Figure S3. ^{1}H-^{1}H COSY characterization of **PV1** alkoxyamine (A) and **PV2** alkoxyamine (B), recorded in DMSO-d_6.

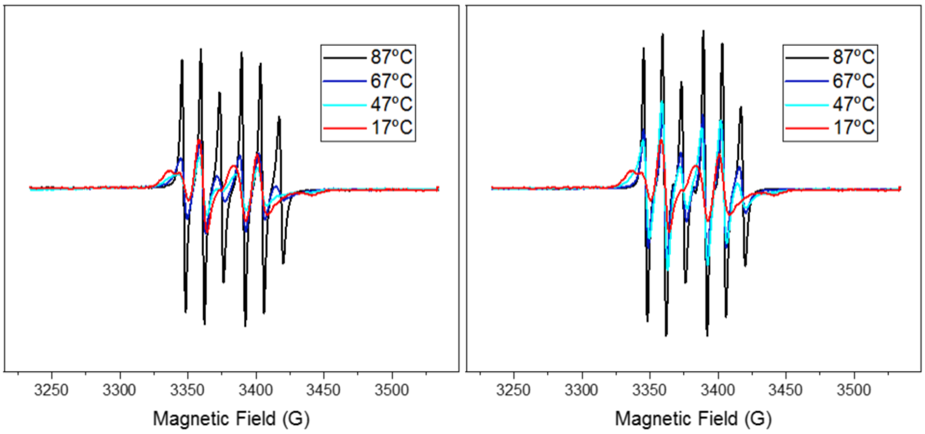

Figure S4. Free radical intensities versus temperature obtained by electron paramagnetic resonance (EPR) for both PV1 (left) and PV2 alkoxyamines (right), respectively.

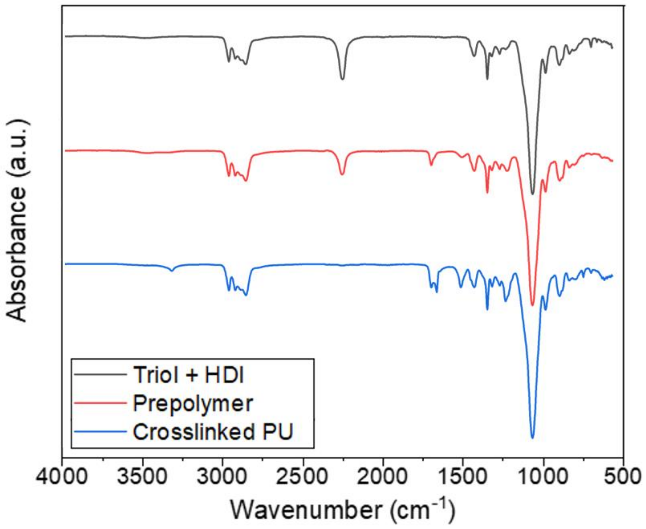

Figure S5. FTIR characterization of blank PU thermoset (**HDO PU**) (1,6 Hexanediol as chain extender). Black trace corresponds to the initial mixture of polypropylene glycol (PPG) and 1,6 Hexamethylene diisocyanate (HDI). Red trace corresponds to the tris-isocyanate terminated prepolymer and blue represents the final cured material after reacting with 1,6 Hexanediol.

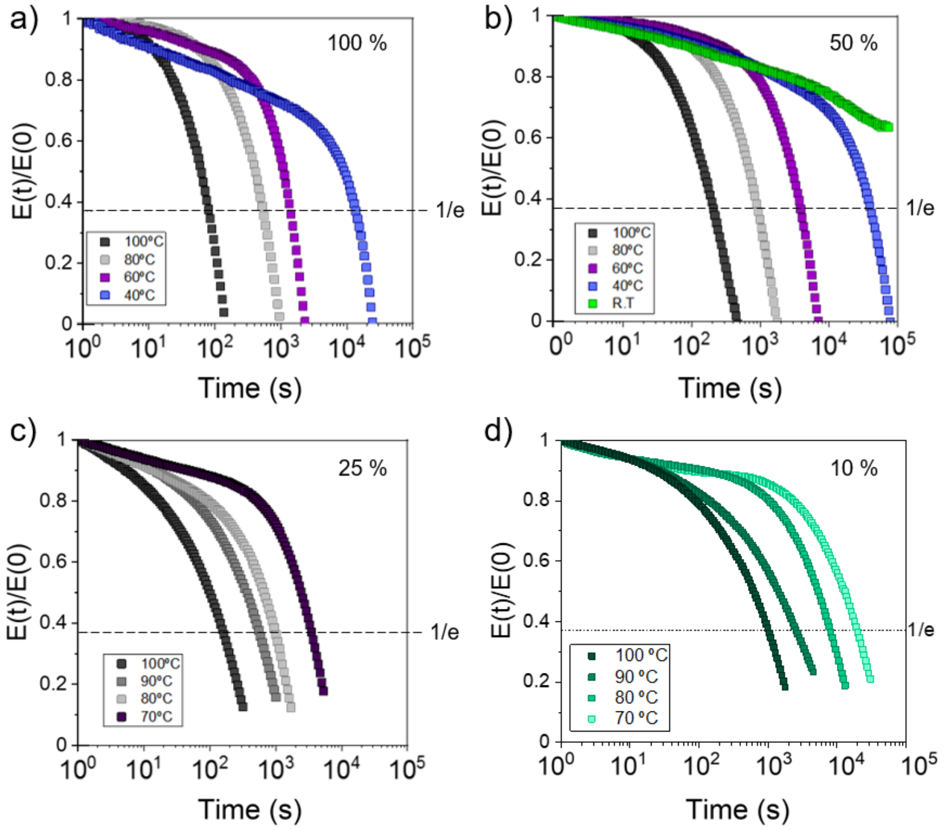

Figure S6. Stress-relaxation measurements of PU films measured at different temperatures containing different ratios of **PV2** a) 100 %, b) 50 %, c) 25% and d) 10 %.

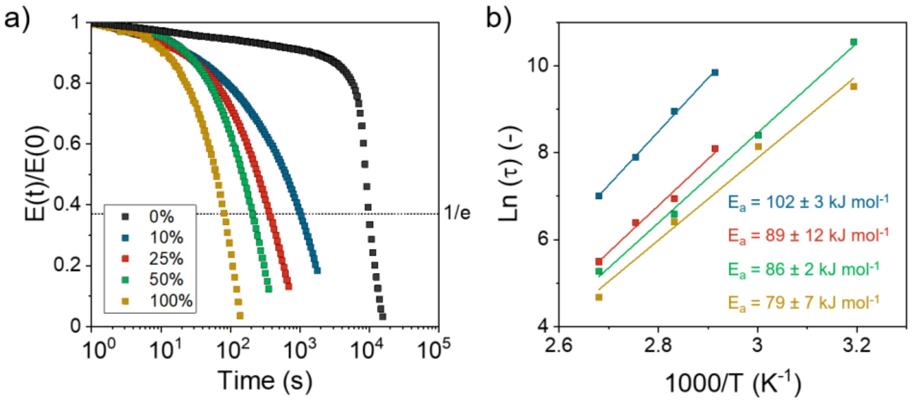

Figure S7. a) Influence of the alkoxyamine concentration in the relaxation time at 100 ºC. b) Arrhenius plot of characteristic relaxation times of cross-linked PU with different amounts of **PV2** alkoxyamine and their respective activation energies (E_a) (black, 0%; blue, 10 %; red, 25 %; green 50 % and yellow, 100 %).

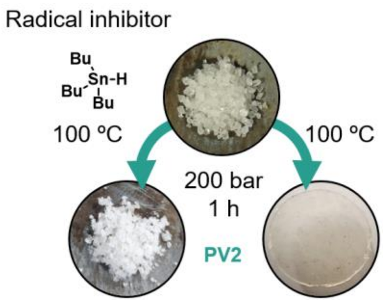

Figure S8. Reprocessing attempt using 5 equivalents of tributyl tin hydride, a radical inhibitor (left) vs reprocessing without additives for PU network containing 50 % of PV2.

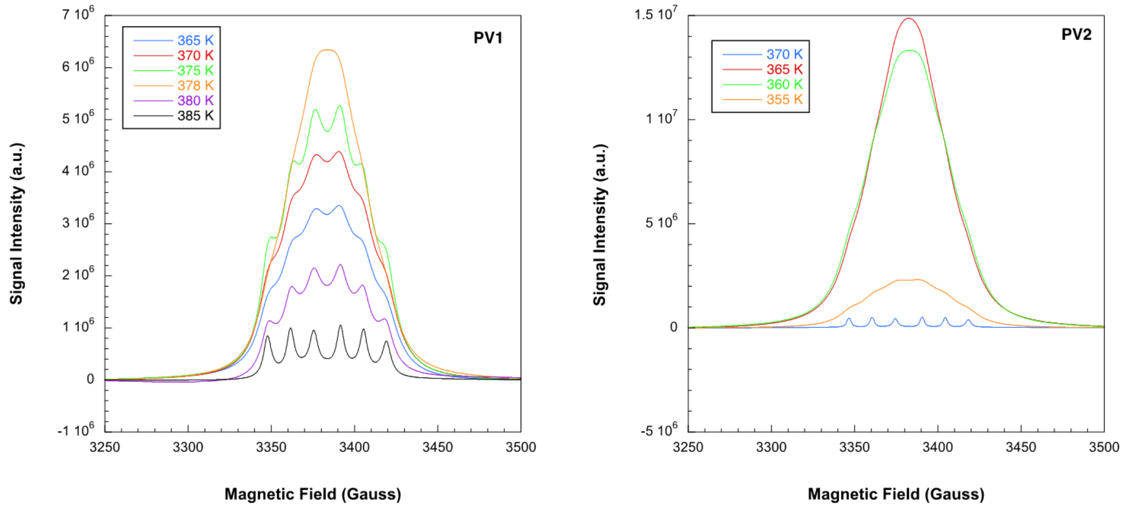

Figure S9. Free radical intensities versus temperature obtained by electron paramagnetic resonance (EPR) for both PV1 (up to 385 K) (left) and PV2 (up to 370 K) (right), respectively.

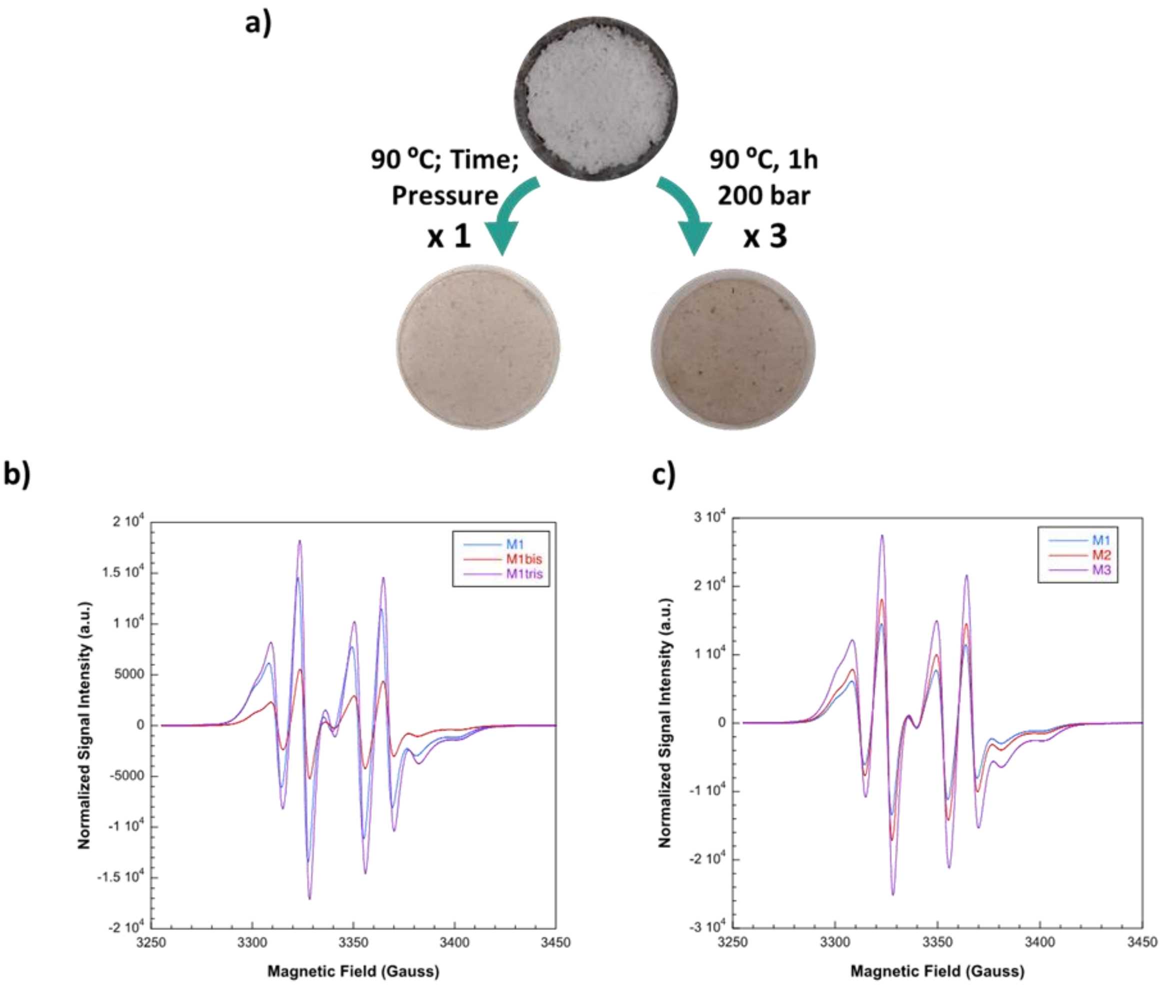

Figure S10. a) Visual images of reprocessing samples of PU with 50% of PV2. Left, one reprocessing at different conditions of pressure and temperature (M1:M1bis:M1tris) and right, 3 reprocessing cycles at 90 ºC for 1h. b) EPR analysis of 3 samples reprocessed at 3 different conditions (M1:M1bis:M1tris). c) EPR experiments of the same sample after 1 cycle of reprocessing (M1) after 2 cycles of reprocessing (M2) and after 3 cycles of reprocessing (M3) at 90 ºC for 1h each.* M1:90 ºC, 1h, 200 bar; M1bis: 90 ºC, 30 min, 200 bar; M1tris: 90ºC, 30 min, 500 bar

The PPG polyol, named Desmophen® 4042 BT, exhibits the following characteristics as specified in the product datasheet:

Hydroxyl number of 44.5 ± 1.5 mg KOH/g.

To determine the M_n, we conducted GPC analysis:

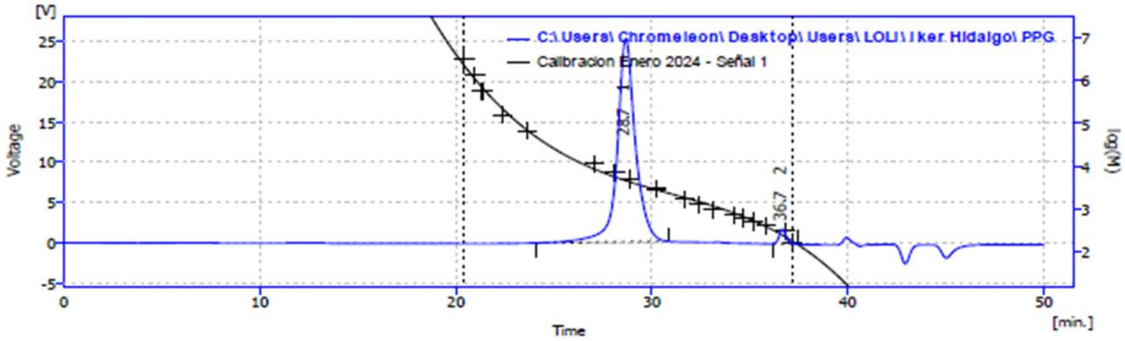

Tabla de resultados (Calibracion Enero 2024 - Corrección del flujo-Univelsal ancha. -
C:\Users\Chromeleon\Desktop\Users\LOLI\Iker Hidalgo\PPG)
Ecuación: Y = - 0.00139*X^3 + 0.12972*X^2 - 4.15041*X + 48.8689
Coeficiente de correlación: 0.9975818

	Tr Max	Mn	Mw	PD	Área [mV.s]	Area [%]
1	28.67	4482	4706	1.0500	1625.77	97.28
2	36.67	244	246	1.0112	45.38	2.72

Figure S11. GPC analysis of the PPG polyol in THF.

Adjustments are necessary for the M_n obtained via GPC, considering the calibration was conducted using Polystyrene. The M_n of PPG, a Poly(Propylene oxide), is determined using the following equation:[1]

$$logM_a = \frac{logK_{PS} - logK_A + (\alpha_{PS} + 1)logM_{PS}}{\alpha_A + 1}$$

Where:

M_a is the unknown M_n (the one of the PPG);

K_{PS} and α_{PS} are the Mark-Houwink parameters for Polystyrene (in THF);

K_A and α_A are the Mark-Houwink parameters for Poly(Propylene oxide) (in THF);

M_{PS} is the M_n of the Polystyrene (found via GPC)

Substituting the values, the equation yields:

$$logM_a = \frac{log11 \cdot 10^{-3} - log12.9 \cdot 10^{-3} + (0.725 + 1)log4482}{0.75 + 1}$$

Thus, $logM_a$ results to be 3630 g/mol.

With the M_n value obtained, the functionality can be determined:

$$\text{g (of KOH to neutralize 1 mole of polyol)} = 0.045\ \text{g} \cdot 3630\ \text{g} = 0.162\ \text{g}$$

$$\text{Functionality (moles of KOH)} = \frac{0.162\ g\ (KOH)}{56.1056\ g/mol(KOH)} = 2.9\ mol$$

which can be approximated to 3.

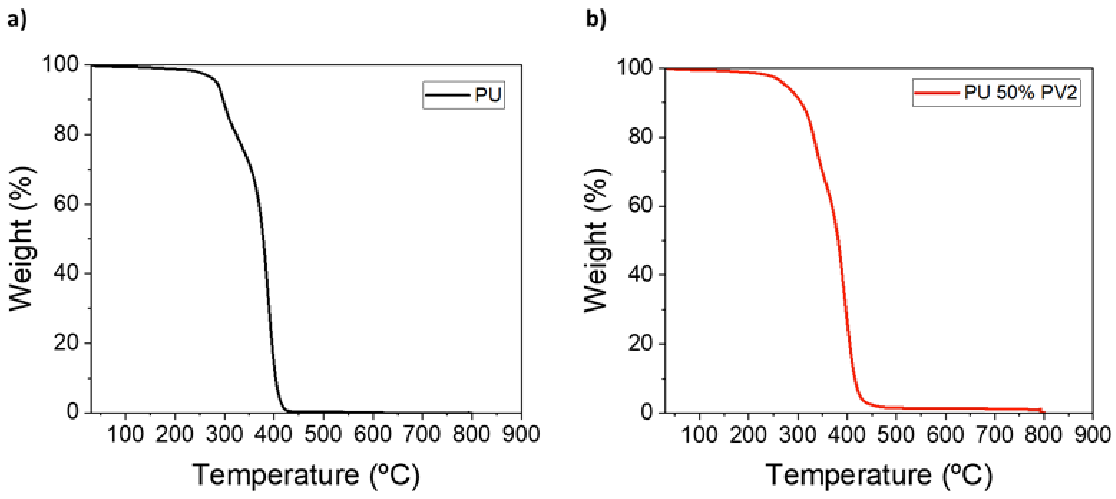

Figure S12. TGA analysis of the PU crosslinked polymer after treating it in at 80 °C for 24h. a) TGA analysis of the PU pristine. b) TGA analysis of the PU containing 50% of PV2.

Gel content

The gel content (insoluble content) was determined according to previously reported procedures.[2,3] The method measures the fraction of a polymer sample that is insoluble in a certain solvent under reflux conditions. For determination of the insoluble content, the dry sample was extracted in boiling THF for 24 h using Soxhlet apparatus. After extraction, the samples were dried in a vacuum oven at 65 °C for 24 h and weighed. The gel content was calculated according to the following equation:

$$X_{gel} = \frac{m_{gel}}{m_0} \cdot 100$$

Where m_{gel} is the dried insoluble fraction or residual mass and m_0 is the initial mass.

Table 1. Calculated values of gel content uf PU-PV1 and PU-PV2 samples before and after reprocessing.

SAMPLE	GEL CONTENT (%)
PU-PV_1 (before reprocessing)	93.6
PU-PV_1 (after reprocessing)	93.3
PU-PV_2 (before reprocessing)	92.9
PU-PV_2 (after reprocessing)	92.7

The gel content did not change significantly between the pristine (non-reprocessed sample) and after the first reprocessing.

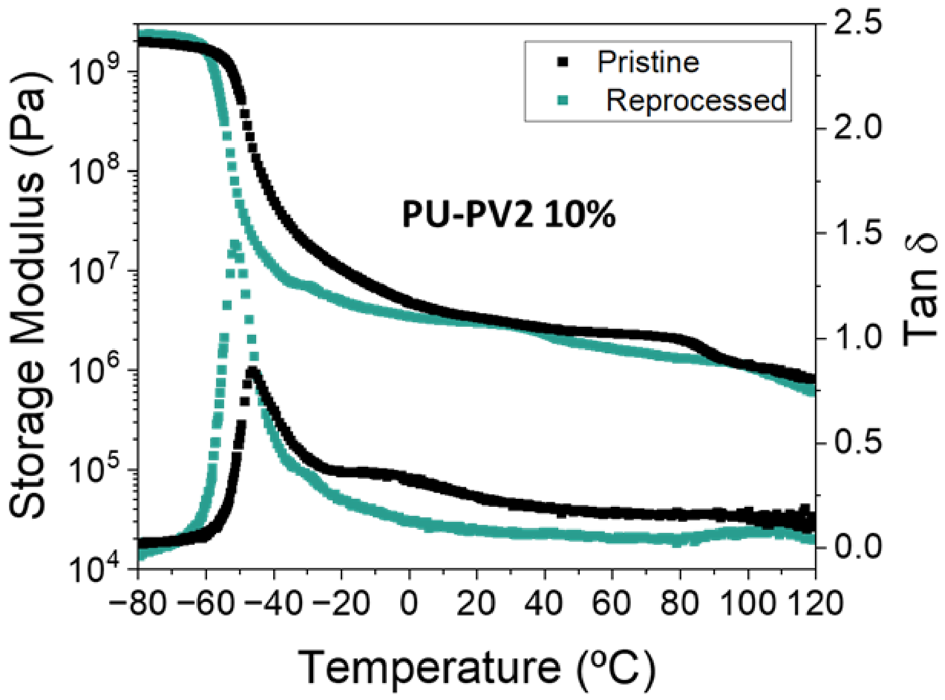

Figure S13. Dynamic mechanical analysis (DMA) analysis of the PU containing the 10% of PV2, before reprocessing (in black) and after reprocessing (in green). The reprocessing conditions were the following: 45 minutes; 90 °C; 400 bar.

Density Functional Theory (DFT) calculations

All geometry optimizations were carried out within density functional theory (DFT) using the M062X functional *(Y. Zhao and D. G. Truhlar, Theor. Chem. Acc., 2008, 120, 215–241)* combined with the 6-31+G(d,p) basis set. *(W. J. Hehre, R. Ditchfield and J. A. Pople, J. Chem. Phys., 1972, 56, 2257–2261)* To confirm that the optimized structures were minima or transition states on the potential energy surfaces, frequency calculations were carried out at the same level of

theory. These frequencies were then used to evaluate the zero-point vibrational energy (ZPVE) and the thermal corrections, at T = 298.15 K, in the harmonic oscillator approximation. Single-point calculations using the 6-311++G(2df,2p) basis set *(R. Krishnan, J. S. Binkley, R. Seeger and J. A. Pople, J. Chem. Phys., 1980, 72, 650–654)* were performed on the optimized structures in order to refine the electronic energy. Solvent effects, in THF, have been estimated using the polarizable continuum model (PCM) approach *(M. Cossi, V. Barone and R. Cammi, Chem. Phys. Lett., 1996, 255, 327–335; E. Cancès, B. Mennucci and J. Tomasi, J. Chem. Phys., 1997, 107, 3032–3041; V. Barone, M. Cossi and J. Tomasi, J. Chem. Phys., 1997, 107, 3210–3221; V. Barone, M. Cossi and J. Tomasi, J. Comput. Chem., 1998, 19, 404–417).* All the calculations were performed with the Gaussian 16 suite of programs *(M. J. Frisch, G. W. Trucks, H. B. Schlegel, G. E. Scuseria, M. A. Robb, J. R. Cheeseman, G. Scalmani, V. Barone, G. A. Petersson, H. Nakatsuji, X. Li, M. Caricato, A. V. Marenich, J. Bloino, B. G. Janesko, R. Gomperts, B. Mennucci, H. P. Hratchian, J. V. Ortiz, A. F. Izmaylov, J. L. Sonnenberg, D. Williams-Young, F. Ding, F. Lipparini, F. Egidi, J. Goings, B. Peng, A. Petrone, T. Henderson, D. Ranasinghe, V. G. Zakrzewski, J. Gao, N. Rega, G. Zheng, W. Liang, M. Hada, M. Ehara, K. Toyota, R. Fukuda, J. Hasegawa, M. Ishida, T. Nakajima, Y. Honda, O. Kitao, H. Nakai, T. Vreven, K. Throssell, J. A. Montgomery Jr., J. E. Peralta, F. Ogliaro, M. J. Bearpark, J. J. Heyd, E. N. Brothers, K. N. Kudin, V. N. Staroverov, T. A. Keith, R. Kobayashi, J. Normand, K. Raghavachari, A. P. Rendell, J. C. Burant, S. S. Iyengar, J. Tomasi, M. Cossi, J. M. Millam, M. Klene, C. Adamo, R. Cammi, J. W. Ochterski, R. L. Martin, K. Morokuma, O. Farkas, J. B. Foresman and D. J. Fox, Gaussian 16, Revision B.01, Gaussian, Inc., Wallingford CT, 2016)*

Bibliography

1 J.E. Mark; Polymer Data Handbook; 1999

2 M. Jaunich, M. Böhning, U. Braun, G. Teteris and W. Stark, *Polymer Testing*, 2016, **52**, 133–140.

3 H. A. Khonakdar, S. H. Jafari, A. Haghighi-Asl, U. Wagenknecht, L. Häussler and U. Reuter, *Journal of Applied Polymer Science*, 2007, **103**, 3261–3270.
